# Risk Factors and Leadership in a Digitalized Working World and Their Effects on Employees’ Stress and Resources: Web-Based Questionnaire Study

**DOI:** 10.2196/24906

**Published:** 2021-03-12

**Authors:** Anita Bregenzer, Paulino Jimenez

**Affiliations:** 1 Institute of Psychology University of Graz Graz Austria

**Keywords:** digitalization, leadership, new ways of working, resources, stress

## Abstract

**Background:**

In today’s world of work, the digitalization of work and communication processes is increasing, and will increase even further. This increase in digitalization at the workplace brings many new aspects of working life to light, such as working in virtual teams, mobile working, expectations of being constantly available, and the need for support in adapting and learning new digital tools. These changes to the workplace can contain risks that might harm the well-being of employees. Leaders can support the well-being of their employees in terms of protecting and replenishing their work-related resources to cope with critical work demands. This so-called health-promoting leadership could serve as a buffer between risk at the workplace and critical outcomes, such as stress, by amplifying work-related resources.

**Objective:**

This study’s aims were twofold. First, we wanted to investigate if risk factors related to higher digitalization at the workplace can be identified and if these risk factors have an impairing effect on the well-being of employees (eg, higher stress and lower resources). Second, we wanted to investigate if the health-impairing effects of these risk factors can be reduced by health-promoting leadership.

**Methods:**

A total of 1412 employees from Austria, Germany, and Switzerland took part in this online study and provided information on their perceived risks at the workplace, their leaders’ health-promoting behaviors, and their work-related stress and resources.

**Results:**

The results of a hierarchical regression analysis showed that all four risk factors of digital work (distributed team work, mobile work, constant availability, and inefficient technical support) were related to higher stress at the workplace. In addition, distributed team work and inefficient technical support were associated with lower work-related resources. A possible buffer effect of health-promoting leadership between these risks and employee well-being was visible for inefficient technical support. In particular, in the case of having fewer support opportunities in learning and using digital tools, leaders could weaken the potential critical effects on stress. As for the other risk factors, leaders might engage in a different leadership behavior to improve their employees’ well-being, as the physical distance between leaders and employees in virtual team work or mobile work could make health-promoting leadership more difficult.

**Conclusions:**

In a digitalized working world, solutions are needed to create working conditions that benefit employees. The results of this study strongly support the importance of investigating risk factors associated with an increase in digitalization at the workplace in addition to traditional risk factors. As for leadership, leaders need to show leadership behavior adapted to a digitalized workplace in order to reduce employee stress and increase work-related resources.

## Introduction

### Background

In the past years, the digitalization of the workplace has been studied more as a phenomenon relevant to a small number of people than as an important and necessary step to improve the working world. Digitalization is currently affecting many areas and will continue to do so in the future, so the effects of digitalization in the workplace must be studied more closely in relation to work processes within the company and in relation to the well-being and performance of employees. Digitalized work brings many new aspects of working life to light, such as working in virtual teams, mobile working, blur between leisure and work, expectations of constant availability, and the frequent need to adapt to digital changes and learn new digital tools [1,2]. Organizations must be able to react adequately to these changes in order to minimize possible critical effects at individual and team levels (eg, stress, engagement, and performance). Owing to the speed at which digitalization is entering the current world of work, solutions are needed just as quickly, as organizations then can prepare their employees optimally for the newly emerging forms of work.

The topic of digitalization of the working world is currently experiencing an upswing in scientific research, especially under the term “new ways of working” (NWW). NWW describes changes to the workplace that take place in the following four aspects: physical workplace, information and communication technology (ICT), organization and management, and work culture [3]. For example, an important aspect of NWW is having more flexibility in deciding when and where employees can work, as well as using ICTs, such as email, smartphones, and videoconferences. It is expected that those aspects should lead to more efficient work processes [4]. Research in this area has focused strongly on the positive effects of new working forms on employees, such as higher engagement and performance [5,6]. However, there is evidence that these new forms of work also have critical effects on employees, such as fatigue and exhaustion [4,7].

Research in this area is important to highlight the risks of a nonoptimal design of a digitalized workplace. However, there is currently a lack of information on how the company and its employees can benefit optimally from increased digitalization of the workplace. One solution to improve working conditions for employees is leadership. Leaders can change their employees’ working conditions and thus impact their employees’ health by managing and allocating resources at the workplace [8]. More specifically, the concept of *health-promoting leadership* includes leadership behaviors that aim at providing resourceful working conditions for employees [9]. This in turn can reduce the negative consequences of critical working conditions such as stress [10]. Leaders can increase resources at the workplace, for example, by specifically supporting the community within the team or by giving their employees possibilities to participate in important decisions.

This study’s aims were twofold. First, we wanted to investigate if risk factors related to a higher digitalization at the workplace can be identified. With the term “risk factors,” we mainly followed the definition of mental risk factors (according to ISO 10075 [11]), which can have an impairing effect on the well-being of employees (eg, higher stress and lower resources). Second, we wanted to investigate if health-promoting leadership moderates the relationship between risk factors of digitalization at the workplace and employees’ stress and resources. More specifically, it is of interest if the health-impairing effects of certain risk factors can be reduced by health-promoting leadership. To our knowledge, the role of health-promoting leadership in workplaces with increasing digitalization has not yet been addressed directly in research.

### Digital Working World and Effects on Employee Well-Being

The working environment is an important context factor at the workplace that affects the health of employees. Being exposed to a critical working environment with high risks might result in negative psychological states that can negatively affect the individual’s behavior at work [12]. These risks can be associated with the physical environment, the organizational and social environment, or the task itself [11,13]. The aim is to design the workplace in such a way that risk factors are minimized or at least the impairing health effect of these risk factors is reduced with specific interventions. However, the traditional working environment has changed through the application of ICT, and “new” working forms have emerged, such as virtual teams and mobile telework [14,15]. These changes are accompanied by new risks in the workplace, and the potential harmful effects on employee well-being have to be examined more closely.

Research in the field of NWW seems to highlight the positive aspects of a digitalized workplace. ten Brummelhuis et al [4] defined NWW as “…a work design in which employees can control the timing and place of their work, while being supported by electronic communication.” Indeed, research shows that when employees experience more freedom in managing one’s own time (ie, home office), the work is experienced as less stressful for employees [16]. However, NWW can also impact the employee’s well-being negatively, such as having more blurred work-home boundaries, more fatigue, and higher mental demands [7]. Research also indicates that NWW might decrease resources such as autonomy. In a study conducted by van Steenbergen et al [16], employees worked in an organization where they could choose to work at home or at the office. However, the organization seemed to prefer work from home, and this preference for a home office might have been expressed by the company in such a strong way that employees experienced a lower feeling of autonomy.

These findings show that positive effects of NWW should not be expected automatically. On the contrary, NWW might include risks that could lead to harmful effects such as higher employee stress [7]. In a systematic review, the authors outlined the positive and negative aspects of NWW with ICT-enabled workers who were flexible in their work [7]. They found that factors, such as geographically distributed team work (“virtual teams”), time- and location-independent work (“mobile working”), and use of information technology at work, might have negative psychological impacts on the well-being of employees and should be addressed when NWW is implemented. Distributed teamwork or mobile work and increased digital communication are also related to a feeling of having to be constantly available, which can also have a negative impact on well-being [1]. Other factors related to NWW, such as higher flexibility, access to organizational knowledge, and independent management of output, are mostly positive factors that benefit the well-being of employees [7]. However, research should focus more on risks to help organizations adequately address these risks at the workplace. The dimensions of NWW that include possible risks and their relationship with the stress of employees are described in detail below.

### Geographically Distributed Team Work

Geographically distributed team work (referred to as “distributed team work” henceforth) has already been extensively investigated in the past under the term “virtual teams” [14]. In virtual teams, “…teams work together over time and distance via electronic media to combine effort and achieve common goals” [17]. Distributed team work has advantages as well as disadvantages. The advantages include reduced travel time and costs, being independent from time and place, including physically disadvantaged employees in the team, and working in a diverse heterogenous team [18]. However, the disadvantages have been studied in more detail. Owing to the geographical distance of team members, it is difficult to form group cohesion, which is why communication is less frequent and conflicts can occur more often than in face-to-face teams [19,20]. Furthermore, with the use of virtual media, important auditory and visual cues are not perceived sufficiently, which makes communication more difficult [21].

The critical effects of distributed team work on employees’ stress have already been studied [22,23]. For example, virtual teams have more conflicts than traditional face-to-face teams and have difficulties in applying conflict management strategies [21]. More conflicts within the team result in more stress [24]. Stress can occur because of the excessive use of virtual communication media as well (eg, email flood [23]).

### Mobile Work

The use of mobile devices allows employees to work in a distributed team and to work independent of time and location, because messages can now be sent and received from anywhere and at any time. Thus, high flexibility in the daily work routine can be achieved [25]. Work can be done from one’s own home, from an external location, or from another continent. For this kind of work, the term “mobile telework” is commonly used in the literature, which is described as “work at a range of locations, spending regular and significant amount of time away from any office or home location” [26].

Mobile telework can differ from one job to another. There are jobs in which the place of work changes several times a week or day and employees cannot freely choose the work location, for example, work in sectors such as wholesale and retail trade, manufacturing, transportation and storage, information and communication, public administration, and health [27]. The effect of mobile telework on employee well-being can be quite different for those who have control over their working location as compared to that for those who have little say in where they must work [28].

Mobile telework is seen as a resource, especially when you can decide yourself where and when you work [29,30]. However, the physical distance between team members and leaders can reduce the quality of the relationship between employees and leaders [31]. Mobile telework is particularly demanding when the work location is uncertain or when the employees have less flexibility in organizing their work time [32]. Interruptions and distractions can occur more easily in mobile telework than in fixed workplaces (such as offices); for example, interruptions and distractions are more frequent in trains or in public places [32]. In addition, working at multiple locations increases mental demands, such as the feeling of “timeless” continuous work, constant changing of the rhythm of work, and reduced professional and social interaction [15].

### Constant Availability

The use of ICT makes it easier to stay in touch with leaders, colleagues, customers, and family, as contact can now be made anywhere and at any time. This leads to the impression that people are available anywhere and at any time, which can have critical effects on employees’ well-being [33,34].

The expectation of having to be constantly available for work leads to difficulties detaching from work during leisure time and to a stronger work-home interference [35,36]. Especially when the experienced work-home interference is high, using the smartphone for work-related purposes after work has a critical effect on employees’ recovery process [37]. A longitudinal study showed that being constantly available for work increases emotional exhaustion over time [38]. Constantly receiving and checking work-related messages might lead to information overload, as people struggle with managing the inflow of messages [39]. This struggle to keep up with the increasing amount of information leads to higher stress [40,41].

On the contrary, being constantly available can have benefits for employees’ well-being. In a study conducted by ten Brummelhuis et al [4], being constantly available through the use of mobile communication tools was associated with greater engagement. The authors argued that constant availability via email or telephone was associated with greater work flexibility, which was perceived as an advantage by employees. On the other hand, constant availability was also associated with more interruptions at work, which caused more exhaustion among employees.

### Learning and Adapting to Digital Tools

The constant use of ICT for work-related activities raises another point that could be a risk factor for employees’ well-being. In today’s working world, new technologies are developed almost faster than people can learn and use them. The increasing amount and use of ICTs can lead to higher job demands in terms of mental and emotional overload, which might harm the well-being of employees [42]. Thus, the need for support in the use of digital tools and the need to build up competence in handling digital media are growing [43]. In the past, the increasing requirements to be able to handle digital tools were also investigated under the term “technostress.” Technostress is described as the mental stress that employees experience when they are asked to learn and use a new technology [44]. Weil and Rosen [44] found that technostress occurs if people are not taught how to handle technology adequately. Uncertainty about how to deal with new technology and the resulting inefficiency in dealing with modern technologies are currently still important issues in technostress research [45].

If the used technologies change too fast, employees experience difficulties in coping with the changes, which can raise work overload and stress [46]. On the contrary, adapting to new technologies at work might benefit the employees as well. Studies show that having a higher technological demand at work is related to engagement, indicating that learning new technological tools is perceived as challenging [47].

To ensure that technical changes in the workplace are experienced as positive challenges and not as hindrances, it is important that employees are adequately supported in learning and applying these technologies. For example, providing training or guidelines on how to deal with new media and having technical support at work are important for greater well-being at the workplace [48,49]. Social support from supervisors or colleagues is an important factor as well [42]. In the study by Knani et al [50], employees were introduced to a new technology at the workplace, which demanded high learning effort and led to higher emotional exhaustion. The critical effect on emotional exhaustion could reduce when employees experience high support from supervisors and employees. Atanasoff and Venable [51] added that employee-oriented leadership behavior is an important resource that might reduce the negative effects of digitalization, such as stress.

### Digital Workplaces, Leadership, and Resources

Leaders in particular are challenged in a modern working environment. Research in the field of a home office and virtual teams has shown that leadership in a digitalized working environment has different requirements than in traditional work settings [52]. Working in a home office or virtual work in general requires a different role of leadership, in which the manager must lead strongly in an employee-oriented way [53]. An employee-oriented leadership is also preferred in working environments with high demands. According to Wegge et al [54], leader behavior can serve as a buffer between high work demands and critical outcomes, such as stress, by amplifying work-related resources at the workplace. Given the assumption that digitalized workplaces entail high demands, increasing work-related resources through leadership behavior is a particularly important aspect of supporting well-being in the workplace.

Work-related resources play a major role in the relationship between demands and stress [55]. Social resources (social support from colleagues) and task resources (autonomy, the possibility of participation, and the possibility of conducting breaks) are important work-related resources to reduce negative outcomes, such as stress and burnout. A highly digitalized workplace can contain risk factors that might lead to increased demands [46]. In workplaces with high demands, resources could be insufficiently gained, depleted, or even lost, which can cause stress and might increase the risk of getting burnout over time [56].

Maintaining and increasing work-related resources are therefore essential aspects of a health-promoting workplace. Leaders can support their employees in protecting and replenishing their work-related resources to cope with the demands of their work by showing health-promoting leadership behavior [57,58]. Health-promoting leadership is a positive leadership behavior, which enhances the work-related resources of employees. By changing working conditions (such as the health-promoting design of the six areas of work life [59]), it is possible to build up employees’ work-related resources [60]. For example, leaders can ensure that work processes are organized in such a way that employees can cope well with increased workload. Leaders can give their employees opportunities to work autonomously and independently. Rewarding employees is also an essential aspect that can be undertaken by leaders in the form of positive feedback and appreciation. Leaders can strengthen the community in their team by encouraging open communication and mutual support. Acting fairly and paying attention to the values of employees are further aspects of health-promoting leadership [60].

Increasing work-related resources is also essential for a workplace with a high level of digitalization. Atanasoff and Venable [51] assumed that stress due to digitalization is related to lower work-related resources. According to the authors, important resources that should be increased are social support from colleagues, opportunities to participate in the use of technology, and clear information about technology. Therefore, health-promoting leadership could benefit a digitalized workplace as resources are preserved and restored.

The increasing digitalization of workplaces leads to changes in working conditions, which can be risk factors for reduced well-being and performance. Health-promoting leadership can minimize the negative effects of these risk factors by building up enough work-related resources to cope with these risk factors. This way of leadership behavior is described as the “buffer effect,” which means leaders serve as a buffer against high work demands that might be a potential source for stress [54].

### Study Aims and Hypotheses

In this study, we investigated the following four possible risk factors of digital work that could lead to higher stress and lower work-related resources among employees: distributed team work, mobile work, constant availability, and inefficient technical support. First, these four risk factors were examined with regard to their effects on the stress and work-related resources of employees. Second, a possible buffer effect of health-promoting leadership on the relationship of these risk factors with stress and work-related resources was analyzed. This will deepen the understanding of the importance of health-promoting leadership for digitalized workplaces and give an answer to the question of whether leadership behavior can reduce the potential harmful effects of risk factors in digitalized workplaces.

**Figure 1:**
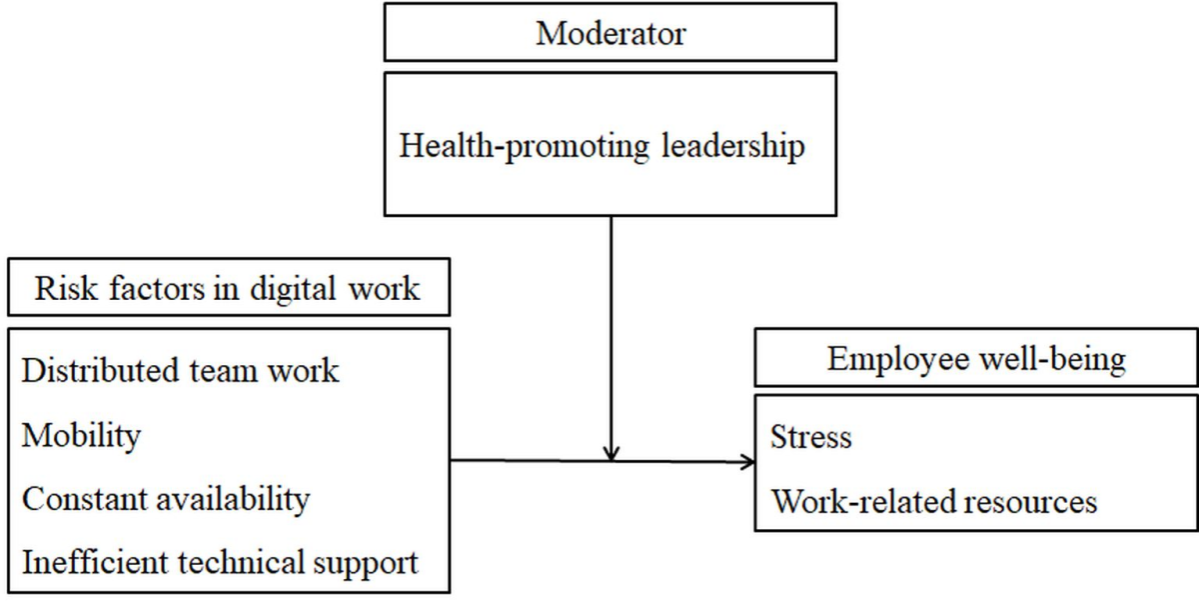
Overall conceptual model of the study.

The following four hypotheses are proposed: (1) H1, risk factors in digital work (distributed team work, mobile work, constant availability, and inefficient technical support) positively relate to employees’ stress; (2) H2, risk factors in digital work (distributed team work, mobile work, constant availability, and inefficient technical support) negatively relate to employees’ work-related resources; (3) H3, health-promoting leadership moderates the positive relationship between the risk factors in digital work and employees’ stress (the relationship is weaker when health-promoting leadership is high); and (4) H4, health-promoting leadership moderates the negative relationship between the risk factors in digital work and employees’ work-related resources (the relationship is weaker when health-promoting leadership is high). Figure 1 summarizes the overall conceptual model of the study.

## Methods

### Participants and Procedures

The study was conducted as an online study with online questionnaires via the online survey platform Questback. The invitation for the online study was sent out in cooperation with a well-known German market research company. The participants of the study were recruited from the company’s online panel. To obtain a heterogenous sample, we set the target male-to-female ratio at about 50:50. The same ratio was used for age (50% for <40 years and 50% for ≥40 years). As the online survey was in German, only German-speaking people were considered for recruitment (ie, persons from Germany, Austria, and Switzerland). The market research company contacted people in the online panel according to these specifications via email. The only criterion for participation in the study was work for at least 10 hours per week. If individuals stated in the questionnaire that they were working less than 10 hours per week, they were filtered out, and on the next page of the questionnaire, they were told that unfortunately they did not belong to the target group. The survey was then closed for this group.

On the first page of the survey, participants were informed about the purpose of the study, the length of the study, and the contact address of the research group. Participation was voluntary, and a small incentive was offered to people who completed all online questions.

Through this procedure, a representative sample of 1412 German-speaking workers in Austria (n=481, 34.06%), Germany (n=720, 50.99%), and Switzerland (n=211, 14.94%), who filled in all online questionnaires, was obtained. In this sample, 56.94% (804/1412) were women and 43.06% (608/1412) were men. The mean age was 41 years (mean 40.77 years, SD 12.30 years). Additionally, 24.36% (344/1412) had a graduate degree. On average, the participants worked 35 hours per week (mean 35.07 hours, SD 11.58 hours).

The participants worked in different business sectors. The majority worked in the service sector (257/1412, 18.20%), followed by health care (192/1412, 13.60%), commerce (167/1412, 11.83%), manufacturing (136/1412, 9.63%), and the public sector (127/1412, 8.99%).

### Measures

#### Risks in Digital Work Scale

In the risks in digital work scale, 10 items measure different work characteristics in a digitalized workplace that could increase demands at the workplace for the following: (1) distributed team work, (2) mobile work, (3) constant availability, and (4) inefficient technical support. The items are written as statements and refer to the last 4 weeks (“How many times have you experienced the following aspects in the last 4 weeks?”). The 7-point scale ranges from 0 (never) to 6 (always). Example items for the four dimensions are shown in Table 1.

**Table 1:** Example items for the risks in digital work scale.

Construct/scale	Sample item
Distributed team work	My colleagues at other locations and I support each other (reversed).
Mobile work	Within a day, my work location changed.
Constant availability	I was available for work in my free time (eg, by telephone or email).
Inefficient technical support	I received support in case of uncertainties in the technical operation of devices, software, and others (reversed).

#### Health-Promoting Leadership

Health-promoting leadership was measured with the health-promoting leadership conditions questionnaire (HPLC) [9], where employees are able to evaluate the frequency of health-promoting leadership from their direct supervisor during the last 4 weeks. In this study, a short version with seven items was used, where each item can be related to one of the following seven aspects of health-promoting leadership: health awareness, workload, control, reward, community, fairness, and value fit. The items are rated on a 7-point scale ranging from 0 (never) to 6 (always). One example item for the dimension community is “In the last 4 weeks, my leader took care that…work is appreciated.”

#### Stress and Resources

The Recovery-Stress Questionnaire for Work (RESTQ-Work) [61] assesses the stress state and experienced resources in the past 7 days/nights. In this study, the short version of the RESTQ-Work (RESTQ-Work-27) with 27 items was used. The items can be assigned to a stress or resource score. The stress score consists of 10 items, and the resource score consists of 17 items. The answer scale is a 7-point scale ranging from 0 (never) to 6 (always). One example item for the stress score is “In the past 7 days/nights…I felt frustrated through my work,” and one example item for the resource score is “In the past 7 days/nights…I had the chance to make suggestions at work.”

### Statistical Analyses

The analyses consist of two parts. First, bivariate correlations showed the relationships between all study variables. Second, a hierarchical regression analysis was used to test the hypotheses regarding the moderator effects of health-promoting leadership on the outcomes of stress and work-related resources. To test the moderating effects of health-promoting leadership, interaction terms between health-promoting leadership and all four risks in digital work variables were computed. Before computing the interaction terms, the variables were mean centered (ie, *z* standardized). For the analyses, SPSS 26.0 (IBM Corp) was used.

## Results

### Descriptive Statistics

Descriptive statistics (means and standard deviations) and reliabilities (Cronbach α) of the study variables are shown in Table 2. Correlations of all study variables are shown in Table 3.

**Table 2:** Descriptive statistics and reliabilities of the study variables.

Dimension	Score, mean (SD)	α
Distributed team work	2.44 (1.53)	.60
Mobile work	1.06 (1.19)	.62
Constant availability	2.19 (1.80)	.71
Inefficient technical support	2.43 (1.66)	.85
Health-promoting leadership	2.99 (1.59)	.93
Work-related resources	3.24 (1.04)	.92
Stress	2.01 (1.30)	.93

**Table 3:** Correlations between all study variables (N=1412).

Dimension			Distributed team work		Mobile work		Constant availability		Inefficient technical support		Health-promoting leadership		Work-related resources		Stress
**Distributed team work**															
	*r*	1		0.10		−0.13		0.58		−0.52		−0.56		0.28	
	*P* value	—^a^		<.001		<.001		<.001		<.001		<.001		<.001	
**Mobile work**															
	*r*	0.10		1		0.29		0.16		−0.09		−0.10		0.22	
	*P* value	<.001		—		<.001		<.001		<.001		<.001		<.001	
**Constant availability**															
	*r*	−0.13		0.29		1		−0.10		0.11		0.12		0.08	
	*P* value	<.001		<.001		—		<.001		<.001		<.001		<.001	
**Inefficient technical support**															
	*r*	0.58		0.16		−0.10		1		−0.51		−0.50		0.26	
	*P* value	<.001		<.001		<.001		—		<.001		<.001		<.001	
**Health-promoting leadership**															
	*r*	−0.52		−0.09		0.11		−0.51		1		0.66		−0.38	
	*P* value	<.001		<.001		<.001		<.001		—		<.001		<.001	
**Work-related resources**															
	*r*	−0.56		−0.10		0.12		−0.50		0.66		1		−0.43	
	*P* value	<.001		<.001		<.001		<.001		<.001		—		<.001	
**Stress**															
	*r*	0.28		0.22		0.08		0.26		−0.38		−0.43		1	
	*P* value	<.001		<.001		<.001		<.001		<.001		<.001		—	

^a^Not applicable.

### Regression Analyses

To test our hypotheses, two step-wise regression analyses were conducted where stress and work-related resources served as the outcomes and the risk factors of digital work and health-promoting leadership served as the predictor variables. In the first step, the risk factors of digital work, including distributed team work, mobile work, constant availability, and inefficient technical support, were entered. In the consecutive second step, health-promoting leadership was entered as the moderator variable. In the third and final step, the interaction terms of the moderating variable health-promoting leadership with the four risk factors were entered. To test if multicollinearity was an issue in our data, we tested the variance inflation factor for all independent variables. All variance inflation factors were below 3 (ranging from 1.02 to 1.70). Thus, multicollinearity was not an issue in our study.

#### Regression Analysis With the Outcome Stress

Table 4 summarizes the regression results for the criterion stress and shows that distributed team work, mobile work, constant availability, and inefficient technical support accounted for 13% of the variance in stress. Distributed team work (β=.19, *P*<.001), mobile work (β=.15, *P*<.001), constant availability (β=.08, *P*=.003), and inefficient technical support (β=.13, *P*<.001) showed significant relationships with stress.

In the second step, health-promoting leadership accounted for an additional 6% of the variance in stress. The relationship with stress was negative (β=−.31, *P*<.001), indicating that high health-promoting leadership is associated with low stress.

In the third and final step, the interaction terms of the moderating variables were entered. The two interaction terms mobile work*health-promoting leadership (β=.11, *P*<.001) and inefficient technical support*health-promoting leadership (β=.07, *P*=.03) were significant. The results did not show a moderating effect of health-promoting leadership for the predictors distributed team work and constant availability. This step accounted for an additional 2% of the variance in stress.

**Table 4:** Results of hierarchical multiple regression analyses for the criterion stress (R2=20.7%).

Step and variable		Stress results^a^							
		B	SE B	β	*P* value	*t* (df)^b^	*F* (df)	*P* value	
**Step 1**							50.880 (4,1407)	<.001	
	Distributed team work	0.25	0.04	.19	<.001	6.32 (1407)			
	Mobile work	0.21	0.04	.15	<.001	5.74 (1407)			
	Constant availability	0.11	0.04	.08	.003	2.94 (1407)			
	Inefficient technical support	0.17	0.04	.13	<.001	4.17 (1407)			
**Step 2**							65.673 (5,1406)	<.001	
	Distributed team work	0.12	0.04	.09	.003	3.01 (1406)			
	Mobile work	0.20	0.04	.15	<.001	5.67 (1406)			
	Constant availability	0.12	0.03	.09	<.001	3.56 (1406)			
	Inefficient technical support	0.04	0.04	.03	.30	1.03 (1406)			
	Health-promoting leadership	−0.40	0.04	−.31	<.001	−10.45 (1406)			
**Step 3**							40.545 (9,1402)	<.001	
	Distributed team work	0.12	0.04	.09	.003	3.02 (1402)			
	Mobile work	0.21	0.04	.15	<.001	5.87 (1402)			
	Constant availability	0.11	0.03	.08	.001	3.23 (1402)			
	Inefficient technical support	0.04	0.04	.03	.30	1.03 (1402)			
	Health-promoting leadership	−0.35	0.04	−.27	<.001	−8.84 (1402)			
	Distributed team work*health-promoting leadership	−0.05	0.04	−.04	.23	−1.19 (1402)			
	Mobile work*health-promoting leadership	0.15	0.04	.11	<.001	4.21 (1402)			
	Constant availability*health-promoting leadership	0.04	0.03	.03	.25	1.15 (1402)			
	Inefficient technical support*health-promoting leadership	0.08	0.04	.07	.03	2.19 (1402)			

^a^Step 1: ΔR^2^=12.64 (*P*<.001); Step 2: ΔR^2^=6.30 (*P*<.001); Step 3: ΔR^2^=1.72 (*P*<.001).

^b^df for *t* values were calculated with the formula N-p-1 (p=number of parameters).

#### Regression Analysis With the Outcome Work-Related Resources

The results for the criterion work-related resources showed that distributed team work, mobile work, constant availability, and inefficient technical support accounted for 37% of the variance in work-related resources (Table 5). Out of these four predictors, distributed team work (β=−.40, *P*<.001), constant availability (β=.05, *P*=.02), and inefficient technical support (β=−.26, *P*<.001) showed significant relationships with work-related resources. Unexpectedly, constant availability showed a low but positive relationship with work-related resources, indicating that being constantly available for work is associated with higher work-related resources. Therefore, only distributed team work and inefficient technical support were negatively related to work-related resources.

In the second step, health-promoting leadership accounted for an additional 15% of the variance in work-related resources. The relationship was positive (β=.47, *P*<.001), indicating that high health-promoting leadership is associated with higher employees’ work-related resources.

In the third and final step, the interaction terms of the moderating variables were entered. The one interaction term of mobile work*health-promoting leadership (β=.04, *P*=.04) was significant. However, the results did not show a moderating effect of health-promoting leadership for the other three predictors.

**Table 5:** Results of hierarchical multiple regression analyses for the criterion work-related resources (R2=51.3%).

Step and variable		Work-related resources^a^						
		B	SE B	β	*P* value	*t* (df)^b^	*F* (df)	*P* value
**Step 1**							204.152 (4,1407)	<.001
	Distributed team work	−0.42	0.03	−.40	<.001	−15.43 (1407)		
	Mobile work	−0.03	0.03	−.03	.17	−1.39 (1407)		
	Constant availability	0.06	0.02	.05	.02	2.39 (1407)		
	Inefficient technical support	−0.27	0.03	−.26	<.001	−9.77 (1407)		
**Step 2**							295.090 (5,1406)	<.001
	Distributed team work	−0.26	0.03	−.25	<.001	−10.39 (1406)		
	Mobile work	−0.02	0.02	−.02	.31	−1.02 (1406)		
	Constant availability	0.04	0.02	.03	.09	1.72 (1406)		
	Inefficient technical support	−0.11	0.03	−.11	<.001	−4.56 (1406)		
	Health-promoting leadership	0.49	0.02	.47	<.001	20.43 (1406)		
**Step 3**							166.282 (9,1402)	<.001
	Distributed team work	−0.26	0.03	−.25	<.001	−10.40 (1402)		
	Mobile work	−0.01	0.02	−.01	.55	−0.60 (1402)		
	Constant availability	0.04	0.02	.03	.11	1.62 (1402)		
	Inefficient technical support	−0.12	0.03	−.12	<.001	−4.79 (1402)		
	Health-promoting leadership	0.48	0.02	.47	<.001	19.68 (1402)		
	Distributed team work*health-promoting leadership	−0.03	0.02	−.03	.28	−1.09 (1402)		
	Mobile work*health-promoting leadership	0.05	0.02	.04	.04	2.04 (1402)		
	Constant availability*health-promoting leadership	−0.03	0.02	−.03	.10	−1.64 (1402)		
	Inefficient technical support*health-promoting leadership	−0.04	0.02	−.04	.08	−1.75 (1402)		

^a^Step 1: ΔR^2^=36.72 (*P*<.001); Step 2: ΔR^2^=14.48 (*P*<.001); Step 3: ΔR^2^=0.43 (*P*=.02).

^b^df for *t* values were calculated with the formula N-p-1 (p=number of parameters).

### Simple Slope Analyses

**Figure 2:**
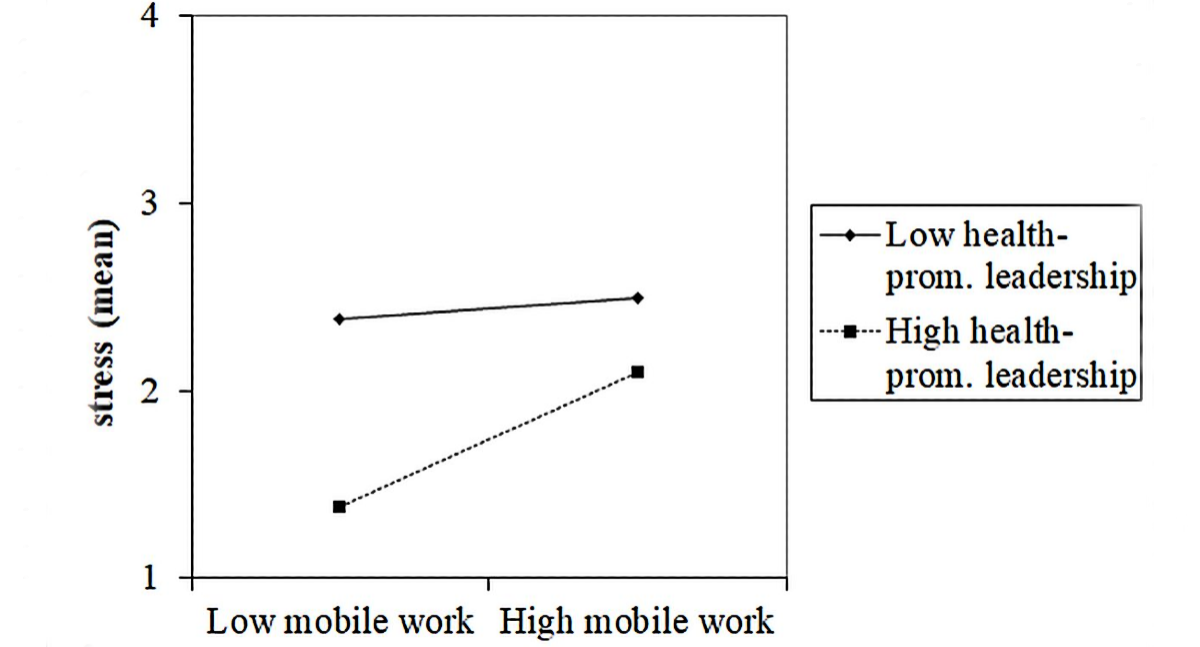
Effect of a two-way interaction between mobile work and health-promoting leadership on stress. prom.: promoting.

**Figure 3:**
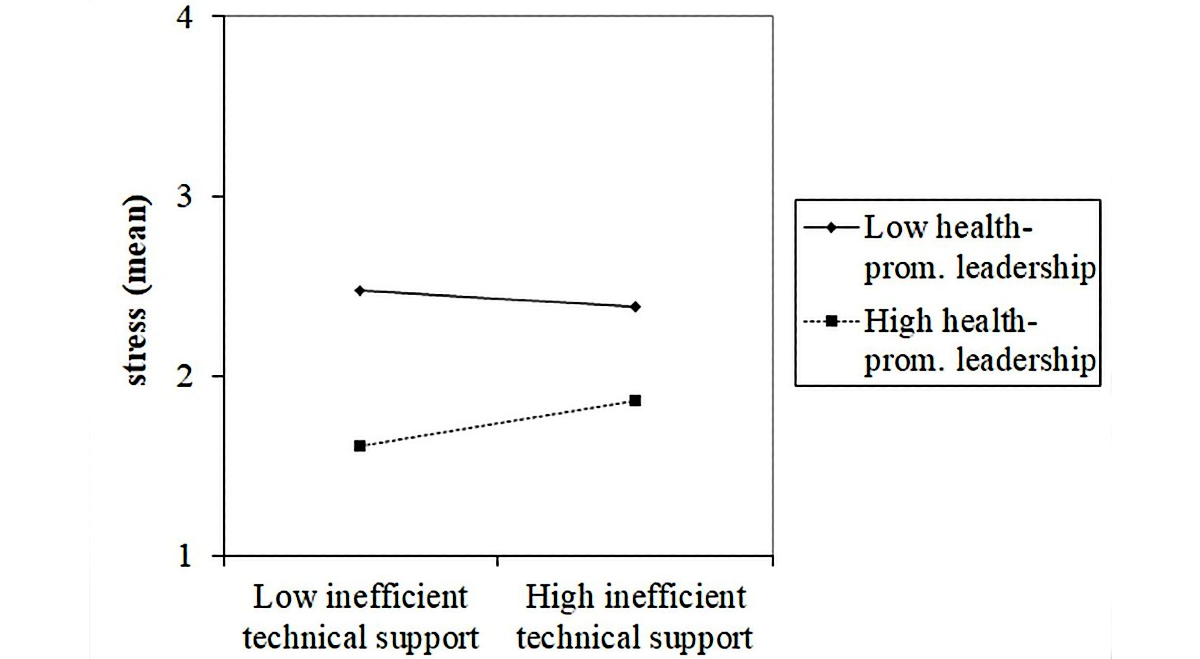
Effect of a two-way interaction between inefficient technical support and health-promoting leadership on stress. prom.: promoting.

**Figure 4:**
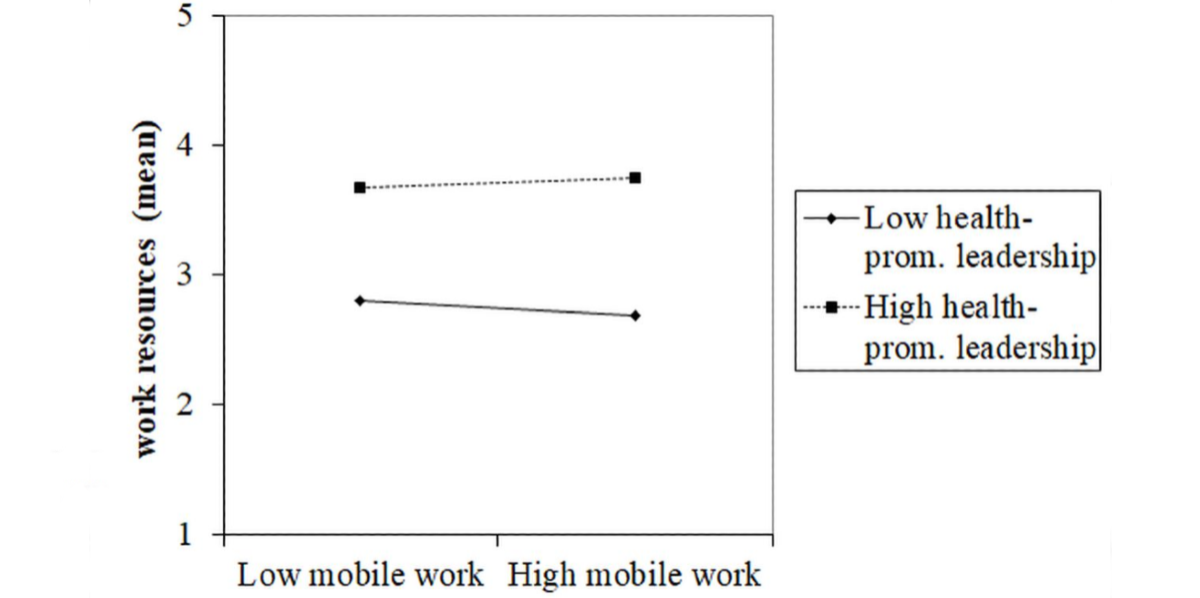
Effect of a two-way interaction between mobile work and health-promoting leadership on work-related resources. prom.: promoting.

In order to investigate the interaction effects, simple slope analyses were conducted for the significant interaction effects (Figures 2-4). The slopes indicated that employees with high health-promoting leadership experienced less stress than employees with low health-promoting leadership. However, employees with high mobile work did not seem to benefit much from health-promoting leadership, as employees with high mobile work and with high health-promoting leadership seemed to have a similar stress level as that in employees with low health-promoting leadership (Figure 2).

As for the risk factor inefficient technical support, having inefficient technical support was related to higher employee stress. Having high health-promoting leadership could buffer this negative relationship, as the stress level of these employees was lower compared to that in employees with low health-promoting leadership (Figure 3).

As for work-related resources, employees with high health-promoting leadership experienced more resources at the workplace than employees with low health-promoting leadership. In terms of low health-promoting leadership, work-related resources were the lowest in the group of employees with high mobile work (Figure 4).

## Discussion

### Principal Results

#### Risk Factors of Digital Work

This study explored the relationships between four risk factors of digital work (distributed team work, mobile work, constant availability, and inefficient technical support) and employees’ stress and work-related resources. In addition, the potential role of health-promoting leadership in reducing the critical effects of digital work was investigated.

The results showed that all four risk factors of digital work (distributed team work, mobile work, constant availability, and inefficient technical support) were related to higher employee stress. In addition, distributed team work and inefficient technical support were associated with lower work-related resources.

This is in line with previous literature on digital workplaces. Distributed team work (or in other words, virtual team work) can lead to higher stress, since team collaboration and team support are difficult in teams with low face-to-face contact [20]. This might be the reason for experiencing higher stress and lower resources in this kind of teamwork, such as lower participation and decision possibilities and lower social support. Inefficient technical support, such as receiving low support in learning and using digital tools, is a critical factor as well, which has potential harmful effects on employees’ well-being. Support options, such as training, peer assistance, and efficient support from the technical department if available, are factors that are relatively easy to implement in the organization and can reduce the critical effects on stress and resources.

The results for the risk factor mobile work showed a positive relationship with stress, which is consistent with previous literature. It has been shown that working in multiple locations increases mental demands, such as more interruptions or distractions, increased feeling of “timeless” continuous work, and constant changing of the rhythm of work [15,32]. However, an effect on work-related resources could not be found in this study. This means that mobile work neither increases nor decreases the work-related resources of employees. Important work-related resources, such as autonomy, decision-making, and participation opportunities, as well as social contact with colleagues, do not seem to be affected by mobile work.

We expected that being constantly available for work via telephone or email would be related to higher stress and lower work-related resources. Indeed, constant availability was related to higher employee stress, indicating that the expectation of having to be constantly available for work can lead to difficulties detaching from work, which harms the well-being of employees [35,36]. Unexpectedly, being constantly available for work showed a low but positive relationship with work-related resources. Another study conducted by ten Brummelhuis et al [4] came to a similar conclusion. In their study, constant availability via email or telephone was associated with greater work flexibility, which is perceived as a resource by employees. The simultaneous perception of increased resources and increased stress at the workplace seems implausible at first, but is actually not a contradiction. In the view of Kallus [62], increased stress and increased work-related resources can occur simultaneously. This seems to be the case with our findings in this study. Being constantly available increases stress, as employees might have difficulties detaching from work. At the same time, employees might experience higher flexibility, which is also associated with work-related resources such as higher autonomy.

However, chronic stress might tax the employees’ resources to the extent that resources are damaged and lost to the point where they cannot be activated anymore [56]. Employees and organizations must therefore always pay attention to a balance between stress and resources. The relationships between constant availability and both outcomes were small though. Further studies are needed to deepen the understanding of the possible critical and beneficial effects of constant availability.

#### Health-Promoting Leadership

Increasing digitalization of the workplace should support employees in their work tasks and not additionally burden them. Leaders play a key role in ensuring that work is designed in a health-promoting way [9]. In this study, we investigated if leaders engaging in health-promoting leadership could act as a buffer between the risks emerging from digital work and critical outcomes of employee well-being.

The results showed interaction effects between mobile work and health-promoting leadership, as well as between inefficient technical support and health-promoting leadership. As for the first interaction effect, the analysis revealed that the combination of low mobile work and high health-promoting leadership was related to low employee stress. This means that having a work location that usually does not change during the week and having a health-promoting leader seems to be the best condition for employee well-being. For employees with high mobile work, the beneficial effect of health-promoting leadership on stress could not be verified. It is possible that the conditions of mobile work make health-promoting leadership behavior more difficult, since the physical distance places special demands on the management and promotion of employees. Therefore, health-promoting leadership cannot buffer the critical effect on stress anymore, as leaders are far away most of the time.

Interestingly, mobile work did not increase or decrease the work-related resources of employees. A potential buffer effect of health-promoting leadership could not be verified. An explanation could be that mobile work itself is perceived as a resource by involving higher autonomy and more decision-making and participation opportunities. Physical distance can also be an obstacle for leaders to build up work-related resources [63].

Further, the results showed an interaction effect between health-promoting leadership and inefficient technical support. Experiencing high support in using digital tools and being led in a health-promoting way seems to be the best combination for employees in regard to stress. With a health-promoting leader and at the same time fewer support opportunities in learning and using digital tools, the stress of employees is high but still below that of employees with low health-promoting leadership. In other words, in the case of experiencing hindrances in learning and using digital tools, leaders can weaken the potential critical effects on stress. This is in line with previous findings, where high support from supervisors helped employees to cope with using new technology at the workplace, which demands high learning effort [42,50].

### Theoretical Implications

Mental risk factors in the workplace that lead to mental stress must be carefully evaluated in each workplace according to international norms like ISO 45001 [64] and especially European laws (eg, the Framework Directive 89/391/EEC [65] and the European Framework for Psychosocial Risk Management, PRIMA-EF [66]). In this study, we were able to show that risk factors in a digitalized work environment must be considered in addition to the commonly evaluated risk factors. Currently, the so-called risk assessment focuses strongly on the following areas: the physical environment, the organizational and social environment, and the task itself (ISO 10075-1 [11]). This study presents the following four possible risk factors that could be included in addition to the aforementioned areas: distributed team work, mobile work, constant availability, and inefficient technical support. We strongly suggest including these risks in current theoretical concepts about risk assessment at the workplace.

In research regarding NWW, possible negative effects of new forms of working are already considered [7]. However, research regarding the positive aspects of NWW still outweighs research regarding the negative effects. Although the advantages of NWW are obvious, such as being flexible regarding working time and location and having higher work-family balance, negative effects are possible if the working conditions are not optimally designed. The results of this study showed that a more critical view of the effects of NWW should be included in research.

In this study, we assumed that leaders who lead in a health-promoting way act as a buffer between work-related demands and employee well-being [54]. The results showed that this buffer effect was visible for one of the four risk factors (inefficient technical support). Although stress among employees increases if they receive little support, explanation, and information when using digital tools, the increase is not as strong if leaders lead in a health-promoting way. For the other three risk factors (distributed team work, mobile work, and constant availability), the results did not indicate a buffer effect.

Employees experience the best working conditions when workplaces have low risks and when health-promoting leadership is high. In a digitalized working world with special risks, such as virtual teamwork, mobile working, and constant availability, it seems that leaders need to show leadership behavior adapted to these working conditions in order to reduce employee stress and increase work-related resources. Research is yet to define such a leadership model that is best suited for digital workplaces. Initial approaches in the field of virtual teams exist, which could serve as a base for such a leadership model [17,67,68]. Nevertheless, the goal should be a broader leadership concept that goes beyond research on virtual teams.

### Practical Implications

In order to remain competitive, many companies are switching to elements of new ways of working, such as home office, mobile work, and increased use of digital media. For successful digitalization of the working process, both the company and individual employees must adapt well to the changed working conditions. Therefore, interventions to support health-promoting digitalization of the workplace have to be developed. In a workplace where digitalization is already well advanced, it is plausible to set digital interventions. For example, the whole process of workplace health promotion can be done digitally, starting with electronic feedback tools to recognize employees’ health states and extending to creating and implementing eHealth tools [69]. The adoption of eHealth tools to promote physical and mental health is an effective way to support employees [70-72]. Organizations can also benefit from eHealth tools by quickly receiving anonymized feedback about the well-being of their employees. In the event of critical feedback, the organization can act to avoid negative consequences, such as stress and burnout.

Leaders in particular must recognize the needs of their employees in a digitalized work environment even more strongly than in traditional work settings and adapt their leadership behavior accordingly. In addition, in the time of COVID-19, the support of leaders plays a much stronger role in reducing the stress for employees [73]. During the COVID-19 pandemic, many employees are working in home offices, and thus, solutions are needed for leaders on how employees can be optimally supported from a distance. The results of this study provide initial insights into the difficulties of leadership in a digitalized work environment. For example, our results indicate that when employees have high mobile work and therefore are locally distant from their leaders, leaders need more support to be able to lead in a health-promoting way. For this kind of work, certain aspects of digitalization can be an advantage, as digital tools can allow leaders to keep close contact with their employees, for example, by using video calls or chat.

### Limitations

This study was a cross-sectional study with the data collected at one measurement point. To determine causality, longitudinal analyses are needed. It seems plausible that risk factors at the organization level affect the well-being of employees and not the other way around. However, it is possible that highly stressed employees perceive certain work characteristics more negatively and thus rate these characteristics as more demanding.

Same-source bias is a possible limitation of the study. As we asked employees to rate risk factors in the organization, we assessed the perceived risk factors from the view of employees. Health-promoting leadership was measured in a similar way. Although most research in the field of work-related risks and work characteristics has been conducted at the individual level (at the level of employees), a multilevel view of work characteristics (eg, bringing together the rating of teams) is a more accurate measurement of risk factors in the organization.

Since we conducted the study through an online panel organization, we did not have any personal information of the participants, such as names and email addresses. Additionally, participation did not entail any obligation or dependency. As a result, we were able to reduce fears of anonymity, and therefore, we can assume that the responses were honest. Of course, there is always the effect that people want to present themselves better than how they are in reality. We cannot completely rule out the possibility that people answered questions about their work environment more critically or less critically. However, we assume that the way the study was conducted reduced this bias.

### Conclusions

The results show that all four risk factors of digital work (distributed team work, mobile work, constant availability, and inefficient technical support) are related to higher stress among employees. As for a possible buffer effect of health-promoting leadership, we found that leaders can mitigate the critical effect of inefficient technical support on stress by showing health-promoting leadership behavior. However, risk factors, such as virtual team work and mobile work, might need a different leadership behavior to reduce the health-impairing effects on employee well-being. The physical distance between leaders and employees in virtual team work and in mobile work might hamper leaders in leading in a health-promoting way. Interestingly, being constantly available for work, including during leisure time, is not as much of a risk as other factors, as employees perceive more work-related resources. More research is needed to identify the conditions under which constant availability has beneficial or impairing effects on the well-being of employees.

## Abbreviations

ICT: information and communication technology
NWW: new ways of working
